# Fragment-Based Lead Discovery Strategies in Antimicrobial Drug Discovery

**DOI:** 10.3390/antibiotics12020315

**Published:** 2023-02-03

**Authors:** Monika I. Konaklieva, Balbina J. Plotkin

**Affiliations:** 1 Department of Chemistry, American University, Washington, DC 20016, USA; 2 Department of Microbiology and Immunology, Midwestern University, Downers Grove, IL 60515, USA

**Keywords:** FBLD, antimicrobial, multidrug resistance, inhibitors of pathogenic bacterial enzymes, phenotypic screens, STD NMR

## Abstract

Fragment-based lead discovery (FBLD) is a powerful application for developing ligands as modulators of disease targets. This approach strategy involves identification of interactions between low-molecular weight compounds (100–300 Da) and their putative targets, often with low affinity (K_D_ ~0.1–1 mM) interactions. The focus of this screening methodology is to optimize and streamline identification of fragments with higher ligand efficiency (LE) than typical high-throughput screening. The focus of this review is on the last half decade of fragment-based drug discovery strategies that have been used for antimicrobial drug discovery.

## 1. Introduction

Bacteria evolved and continue to change in response to environmental stressors including antibiotics. Thus, it is worth realizing that drug resistance is unavoidable, necessitating the development of new strategies to address antimicrobial resistance (AMR). Antimicrobial resistance develops via a number of mechanisms in response to both naturally occurring compounds, i.e., antibiotics, and organically synthesized compounds which are generally referred to as antimicrobials. In order to develop resistance, bacteria have developed multiple mechanisms including drug target modification, efflux pumps, hydrolytic enzyme expression, etc., all of which can be shared with other microbes by the various modes of horizontal gene transmission, i.e., transduction, transformation, and conjugation. [1,2,3]. Multiple approaches to combat drug resistance have been developed, in addition to drugs purified from natural product sources, e.g., soil and plants, and the follow up procedures for chemical modification of these products. One of the latest is fragment-based lead generation (FBLG), or fragment-based lead discovery (FBLD). The focus of this review is on the developments in FBLD over the last five years (2018–2022).

FBLD involves the detection of new inhibitors of known and novel potential drug targets through unique binding site identification, chemistry, or new mechanisms of inhibition that evade existing modes of resistance. FBLD has broad acceptance with a majority of commercial operations utilizing it as part of their generation of lead molecules [4]. The foundational theories of FBLG were implemented in silico around the end of the 1980s [5,6]. However, it wasn’t until the mid-1990s with the introduction of structure activity relationship (SAR) by nuclear magnetic resonance (NMR) that the follow up with experimental screening of fragments towards identification of lead model generation gained widespread usage [7,8]. Utilization of SAR-NMR have propelled the selection and construction of libraries to in-depth identification molecules that show promise with regards to the desired activity in a screening assay, i.e., a hit. For background on other antimicrobial-relevant screening assays for hit discovery and hit-to-lead compound selection, qualification, and subsequent optimization strategies, the reader is directed to the following references [9,10,11,12,13].

A range of biochemical, biophysical, and structural assays are employed to identify ligands that could bind to the designated target. These include, but are not limited to NMR, and more specifically, saturation transfer difference (STD NMR), surface plasmon resonance (SPR), crystallography (X-ray), thermal shift analysis (TSA), microscale thermophoresis (MST), and mass spectrometry (MS), biochemical assays. However, MS and biochemical assays have somewhat limited utility as techniques for ligand identification, thus, are usually used more often in situations where the ligand has binding affinity to the target in the 100 μM range. Each of these methods have their advantages and disadvantages, including solubility requirements for both compound and its ligand. This presents a distinct advantage, since together they provide a robust process for the detection of various dynamic ranges of binding affinity. Often the biophysical/biochemical fragment screens are complimented by virtual screens (VS), i.e., screens in silico [10]. Advances supporting the evolution of FBLD methods in the area of antimicrobial development have been elegantly summarized in a recent review [14].

It is the hope that adaption of the new approach to antibiotic design will make the drugs more resistant to microbial inactivation. However, the distribution of antimicrobial resistance (AMR) is not always predictable and is constantly evolving. For example, a chimeric gene, likely the result of fusion a metallo-β-lactamase gene with a partial aminoglycoside resistance conferring sequence, is the novel gene encoding for the New Delhi metallo-β-lactamase, NDM-1 [15]. These ever-changing targets continue to be one of the challenging areas in drug development.

## 2. Metalloenzymes

### 2.1. Lactamases—Focus on NDM-1

**Figure 1:**
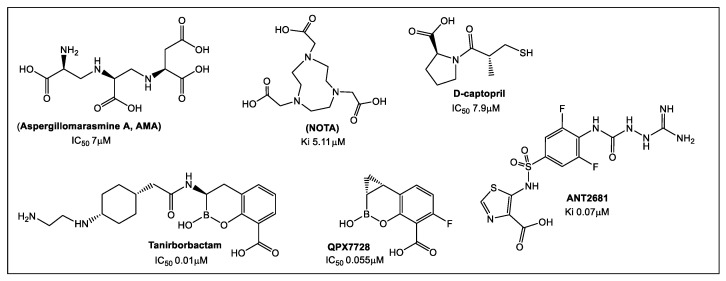
Representatives of the estimated 500 natural and synthetic inhibitors of metallo-β-lactamases.

NDM-1 is clinically an important mechanism of resistance worldwide [16]. Currently, there are no clinically approved metallo- β-lactamase (MBL) inhibitors. Studies on NDM-1 inhibitors have led to the identification of more than 500 potential compounds [17,18]. NDM-1 hydrolysis of the β-lactam ring involves two Zn(II) ions in its active site. Therefore, most of the currently identified inhibitors share common features as chelators of the active site Zn(II) ions. The chelating groups are usually carboxylic acids (e.g., aspergillomarasmine A, AMA; 1,4,7-triazacyclononane-1,4,7-triacetic acid, NOTA) (Figure 1). Additional functional groups include thiols (e.g., D-captopril) and sulfonamide (thiazole carboxylate ANT2681), a preclinical candidate (Ki = 0.07 μM) (Figure 1) [19]. Recently, cyclic boronates such as tanirborbactam (completed phase 3 trials in 2022, Figure 1) [20] and QPX7728 (initiated clinical development in 2021) [21,22,23], which are considered pan-spectrum inhibitors, have demonstrated that NDM-1 inhibition is an achievable bacterial drug target.

**Figure 2:**
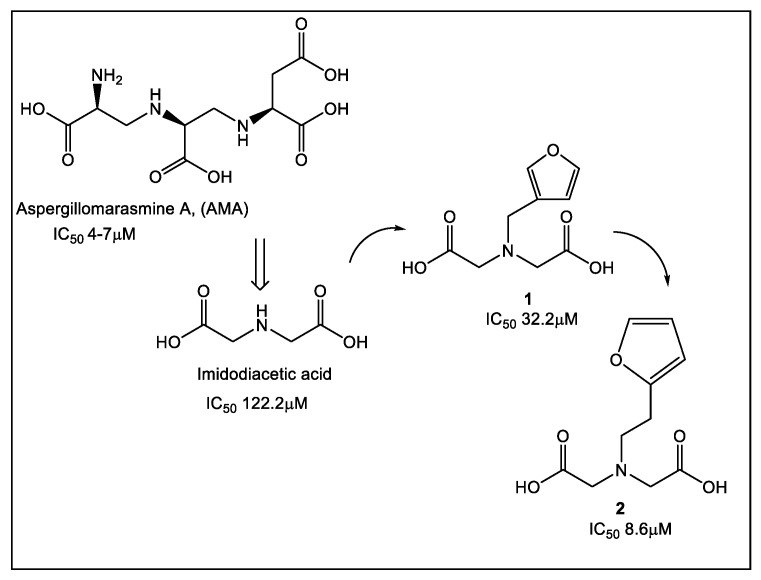
Fragments guide lead derivatization of NDM-1 based on the natural product aspergillomarasmine A (AMA). Iminodiacetic acid (IDA), a simplified analogue of AMA, was successfully derivatized to compound **2** as NDM-1 inhibitor with IC_50_ value similar to that of AMA.

Iminodiacetic acid (IDA) is a novel pharmacophore that has been identified as an NDM-1 inhibitor using the fragments guide lead derivatization FBLD [24]. It was derived from aspergillomarasmine A (AMA), a natural product recognized as a noncompetitive inhibitor of NDM-1 (IC_50_ 4–7 μM). In addition, AMA showed clinical efficacy since it restored meropenem anti-*Klebsiella pneumoniae* NDM-1 activity in a mouse infection model [25,26]. The indiscriminate AMAs metal chelating properties along with the difficult synthesis of AMA’s derivatives has led to the identification of IDA as the metal-binding core of AMA. Based on the IC_50_ (120 μM) of IDA, a fragment-based library was synthesized (Figure 2) and IDA was converted to inhibitor **2** (IC_50_ 8.6 μM, Ki 2.6 μM), which forms a ternary complex with NDM-1 [24].

**Figure 3:**
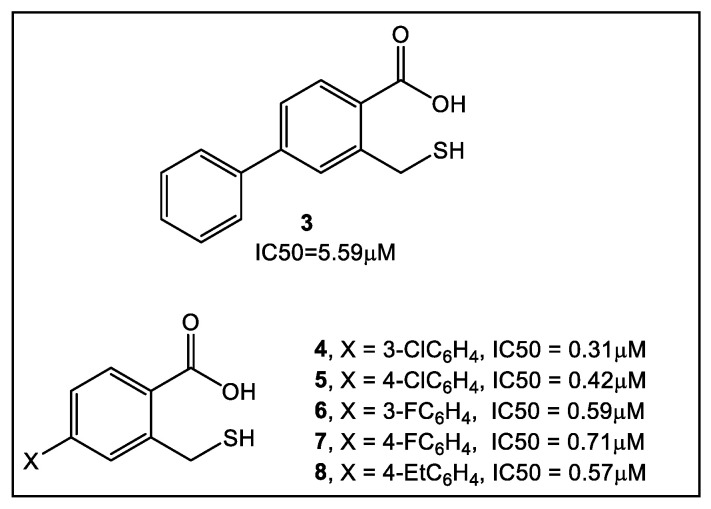
Fragments guiding lead derivatization of NDM-1 inhibitors derived from the natural product captopril making use of the de novo molecular design program SPROUT (a computer program for constrained structural generation). The synthesis of fragment **3** [27] is based on the analysis of a crystal structure of NDM-1 complexed with hydrolyzed ampicillin (PDB ID: 3Q6X) [30]. SPROUT [28] was used to identify active sites for in silico-generated fragments. The sites identified were adjacent to the following: the Lys224 side chain; the Zn-2 metal ion; the nucleophilic hydroxide/water that “bridges” the two zinc ions; and a conserved tryptophan (Trp87) that hydrophobically interacts with the aromatic ampicillin C6 side chain [31], crucial for binding of β-lactams to metallo β-lactamases. SAR also identified several other compounds with activity against NDM-1 in the submicromolar range (4–8) [27].

**Figure 4:**
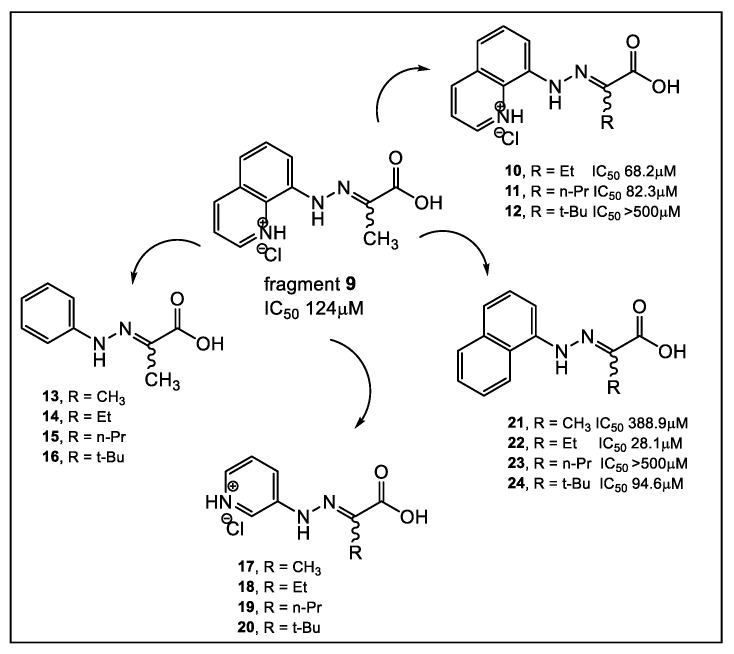
Fragments guide lead derivatization of fragment **9** as inhibitor of NDM-1 predicted by virtual screen of a library of 122,500 fragments. A combination of HTVS, SPR and NMR screening validated the synthesized derivatives of **9** ability to interact with NDM-1 [29].

Similarly, using another natural product, captopril, in silico fragment-based molecular design employing a thiol as a metal chelating motif has led to strong MBL inhibitors [27]. These new inhibitors demonstrated inhibition against NDM-1, as well as verona integron-encoded MBL (VIM) and imipenemase (IMP-1). From the lead fragment **3** (Figure 3), a library of 17 compounds was prepared. The compounds with the most favorable SAR as NDM-1 inhibitors are shown in Figure 3. Using ^19^F NMR to validate the binding of selected fragments to NDM-1, four fragments, including **3** and **4**, were tested for selectivity against two human MBL-fold enzymes involved in DNA repair and DNA cross-link repair, with enzymes 1A and B showing partial selectivity for NDM-1binding. While meropenem alone has an MIC >128 μg/mL against MBL-producing strains of *E. coli* and *K. pneumoniae,* in combination with fragment **3**, meropenem reduced its MIC to 32 and 16 μg/mL, respectively. Interestingly, when tested alone, fragment **3** has no detectable antibacterial activity against the two bacterial NDM-1 producing strains at 100 μg/mL [27]. In addition, these fragments are also inhibitors of Class A and D serine-β-lactamases, albeit weak [27]. In addition to SPROUT [28], other laboratories utilized both Surface Plasmon Resonance (SPR) and STD NMR in searching for a potent NDM-1 inhibitor [29]. Their compound screening used a 122,500-compound library, of which 2500 fragments were obtained from a commercial source, with the rest obtained from an in-house library. Compounds from the latter library that were outside the 150–350 MW fragment range were excluded from the study. After performing high-throughput virtual screening (HTVS) of the library of compounds, followed by SPR validation, 31 fragments were selected based on the data obtained by these two methods, in addition to considering the presence of suitable metal-binding functional groups. This SPR and NMR analysis led to confirmation of the inhibitory activity against NDM-1, albeit weak, of fragment **9** [29]. These findings formed the basis of the synthesis of derivatives of fragment **9** (Figure 4).

Fragments with quinoline (**9**–**12**), naphthalene (**21**, **22**, **24**), and benzene (**13**–**16**) demonstrated medium to weak binding affinities to NDM-1 (Figure 4). Comparing the binding affinities of fragments **9**–**12** and **13**–**16**, Figure 4 indicates that the different R groups: methyl, ethyl, and n-propyl contribute similarly to the affinity, while the branched tert-butyl in the A and D series decrease affinity for NDM-1. Several of these synthesized compounds (**10**, **11** and **22**, Figure 4) demonstrate synergistic antimicrobial activity with meropenem against NDM-1 producing *K. pneumoniae* [29].

**Figure 5:**
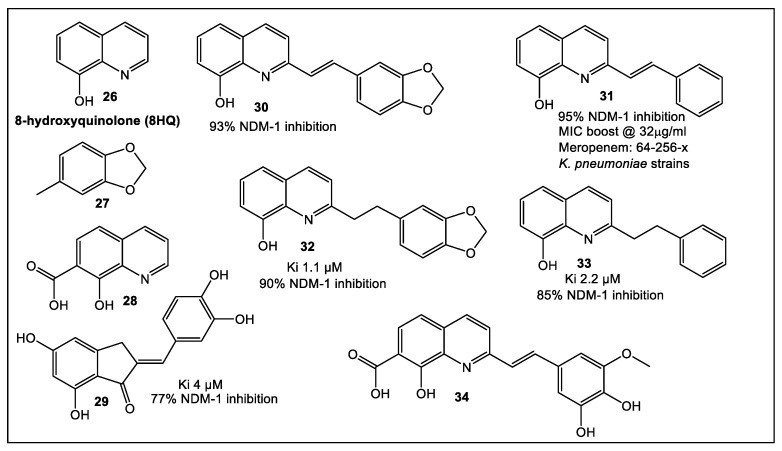
Fragment **26** synthetic analogs. Fragment **26** inhibition of NDM-1 was predicted by virtual library screening (770,000 fragments). To identify and validate fragment interaction with NDM-1, a prepared library of 10 compounds, HTVS, NMR screening and Density Functional Theory (DFT) calculation, and biochemical assays were used [32]. The compounds with estimated optimal putative activity, i.e., Ki and per cent inhibition at 50 mg/mL were determined to be inhibitors of NDM-1 (**29**–**33**) and are shown here. Hit molecule **34** was identified through VS as NDM-1 inhibitor [32].

An alternative approach that combines in silico screening of a very large library of compounds with STD NMR has been used to identify another promising NDM-1 inhibitor [32]. The authors of this study started from a HTVS of a large library of more than 700,000 putative NDM-1 inhibitors. The targeted library was built from fragments obtained from “sliced” hit molecules. This library, in addition to an in-house untargeted fragment library was screened by STD NMR. From the 37 STD NMR identified hit fragments, 10 molecules were synthesized to confirm the abovementioned strategies. The core structure on which further studies were based is fragment **26** (8-hydroxyquinolone, 8HQ, Figure 5) [32]. Fragment **26** (8HQ), nanomolar a broad-spectrum inhibitor against VIM-2 and NDM-1, was initially identified by fragment-based screening of 31 compounds from a commercially available non-specific metal chelator library of fragments (MW = 120–250) through biochemical assays methods [33]. Subsequently, fragment **26** binding to NDM-1 was demonstrated by STD NMR [32]. Fragment **28**, which has fragment **26** as its core structure (Figure 5), has been identified by HTVS [32]. Compound **30** was derived from the structure of hit compound **28**, an HIV-1 integrase inhibitor, which contains two divalent metal cations in its active site [34]. Compound **30** combines fragments **26** and **27**, while compound **31** is based on only one identified fragment (**26**, Figure 5). Further modifications of the initial scaffolds of compounds **30** and **31** gave the corresponding derivatives **32** and **33**, respectively (Figure 5). Compounds **30** and **32** (Ki = 1.1 μM), which combine two high potential fragments, are only marginally better inhibitors of NDM-1 than phenyl analogue **33** (Ki = 2.2 μM). These results indicate that the two-fragment linking strategy is of less benefit in this case; although, a broader analysis may be needed for confirmation. In addition, indenone **29** (Figure 5), which combines two identified fragments, also demonstrated NDM-1 inhibitory activity (Ki = 4 μM) [32]. Moreover, these four 8-HQ derivatives (**39**, **31** and **32**, **33**) can inhibit IMP-1, whereas they are only moderately inhibitory for VIM-2. Compounds **30** and **31**, Figure 5, demonstrated synergistic activity together with meropenem (32 μg/mL) against NDM-1 positive *E. coli* and *K. pneumoniae* clinical isolates. Furthermore, the meropenem MIC was decreased 64- and 256-fold together with compound **31**, in an isolate-dependent manner, in addition to its effect on VIM-2 and IMP-1. MICs of meropenem alone (64 mg/mL) and in combination with inhibitors **31** (0.25 mg/mL, *K. pneumoniae*; 1.0 mg/mL, *E. coli*) and **32** (64 mg/mL, *K. pneumoniae*; 64 mg/mL, *E. coli*) demonstrated superiority of **31** when tested in vitro.

### 2.2. UDP-3-O-acyl-N-acetylglucosamine Deacetylase (LpxC)

**Figure 6:**
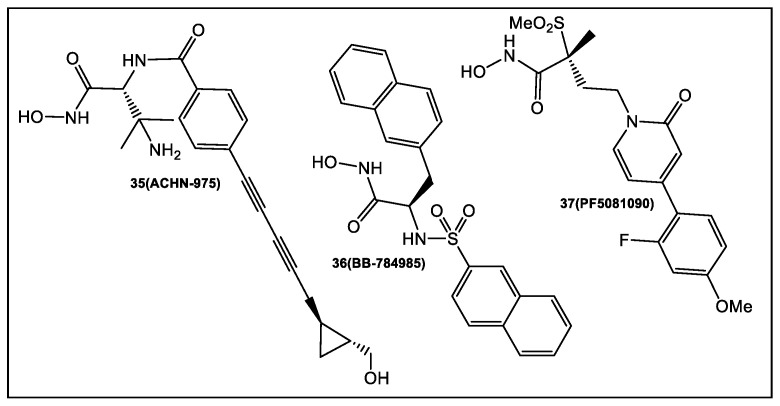
Representatives of hydroxamate-based advanced LpxC inhibitors.

A zinc metalloenzyme, LpxC, catalyzes the first committed step in the biosynthesis of lipid A, a toxic but essential component of the Gram-negative outer membrane [35]. Since LpxC does not have a mammalian homologue, different types of its inhibitors (representatives shown in Figure 6) have been developed, as summarized in references [36,37,38]. Reported compounds’ studies extensively contain a hydroxamate functionality. The hydroxamate functionality combines with the zinc ion on the active site of the LpxC. One of these compounds, **35** (ACHN-9758, Figure 6) [39], was tested clinically; however, the trials were halted, presumably due to off-target-related side effects [39]. These off-target effects may be linked to the hydroxamate moiety which was shown in HDAC inhibitors to be mutagenic [40], presumably due to the nonspecific binding of many matrix metalloprotease (MMP) inhibitors [41].

**Figure 7:**
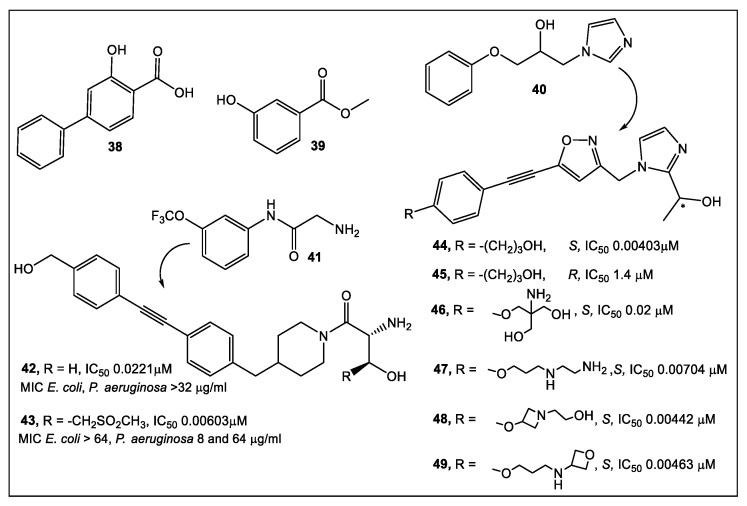
Fragments guide lead **41** derivatization where at low nanomolar concentration (enzyme functional assay) the glycine moiety complexes with zinc. Unfortunately, it has poor antimicrobial activity. Fragments guide lead **40** derivatization can through an imidazole moiety chelate zinc. Imidazole derivative **46**, obtained through structure-guided design, resulted in a 2-(1*S*-hydroxyethyl)- which exhibits inhibition of LpxC at low nanomolar concentrations. In addition, **46** exhibits antimicrobial activity (minimal albumin effects) against *Pseudomonas aeruginosa* at a minimum inhibitory concentration (MIC) of 4 μg/mL [42].

Therefore, the development of fragment-based compounds with moieties different from hydroxamate is highly desirable. Recently, two types of functionalities that bind in the active site of LpxC to the Zn atom via different modes have been reported by researchers from Taisho Pharmaceutical Co., and Vernalis (R&D) [42]. The 1152 compounds of the Vernalis fragment library [42] were screened against PaLpxC in STD, water-LOGSY, and CPMG ligand-observed NMR experiments. Fragments (252) were identified that bound to different LpxC sites by this methodology. Four fragments showed binding across the NMR screening methods, representatives of which are shown in Figure 7.

Of the LpxC bound fragments (Figure 7), molecules competitive with hydroxamate-containing probes (NMR experiments) exhibited dual binding capability by binding to the zinc ion, and the enzyme active site tunnel, as determined by X-ray crystallography. The synthetic efforts have focused on improving the interactions of the new derivatives in both glycine and imidazole fragments (**40** and **41**, Figure 7). Derivative **42** of the glycine series inhibited LpxC with IC_50_ in the nanomolar range (20 nM). However, it did not affect the antibacterial activity (Figure 7). Enzyme activity was improved to the nanomolar range with the addition of a sulfonyl group (**43**, Figure 7). The designed interactions with the protein bound to PaLpxC were confirmed by crystallography [42]. Unfortunately, even this potent derivative of the glycine series in the presence of phenylalanine-arginine β-naphthylamide (PAβN) (an efflux pump inhibitor), demonstrated only minimal antimicrobial activity; therefore, no further development of the glycine series was pursued [42].

After chiral separation of **44** and **45** (Figure 7), it was determined that the *S* enantiomer (>100× enzyme inhibition) has the higher potency. However, the antibacterial activity of *S* enantiomer **44** against a range of Gram-negative bacteria was eliminated by addition of human serum albumin (HSA). Compounds **47** to **49** were produced upon further examination of the solubilizing group (Figure 7). Similar levels of enzyme inhibition were produced by these compounds, with better antimicrobial activity, as compared to **44** and **45**. Of the compounds tested, **49** had the best antibacterial activity across a range of bacterial species, but **46** is the least affected by HSA [42]. Compound **46** is currently undergoing further optimization and determination of its in vivo efficacy [42].

### 2.3. Botulinum Neurotoxins’ Metalloprotease

**Figure 8:**
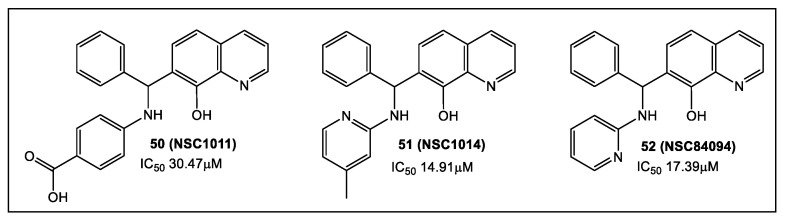
Maximal affinity binding with BoNT/F-LC as shown via SPR was for **51** (NSC1014) (K_D_: 5.58 × 10^-6^). The IC_50_ of **50** (NSC1011), **51** (NSC1014), and **52** (NSC84094) were 30.47, 14.91 and 17.39 μM, respectively (endopeptidase assay). Survival times (mouse model) were extended by **50** (NSC1011) and **51** (NSC1014).

*Clostridium botulinum,* strains of *C. butyricum* and *C. baratii,* which are anaerobic Gram-positive spore-forming bacilli, produce neurotoxins (NT) [43]. These toxins are excreted as a ~150 kDa single polypeptide chain that is cleaved to a heavy chain (HC, 100 kDa) and a light chain (LC, ~50 kDa) by extracellular proteases. Clinical symptoms of neurotoxicity occur post-appearance in the cytosol of LC metalloprotease activity (Zn^2+^ -dependent). Inhibiting metalloprotease activity of different NT serotypes with small molecules has been the focus of numerous studies [43,44,45], including the recent exploration of the quinolinol scaffold [46,47,48,49]. FBLD has been employed for evaluating a 24-compound library, having inhibitors comprised of the 8-hydroxyquinoline (8-HQ) scaffold of botulinum NT serotype F. Compounds were chosen according to computational analysis of ~800 molecules [50]. Selected compounds per the 24 8HQ-based library (fluorescence thermal shift, FTS) were then tested using an endopeptidase assay. The surface plasmon resonance (SPR) based Proteon™ XPR 36 system was utilized for binding affinity analysis. This was followed by in vivo efficacy analysis in a mouse model. The analysis of FTS and endopeptidase assays led to identification of 3, 8HQ fragments—**50** (NSC1011), **51** (NSC1014), and **52** (NSC84094) (Figure 8) as inhibitors of the NT serotype F. The highest binding affinity was shown by SPR studies of **51** (NSC1014, K_D_: 5.58 × 10^−6^) with BoNT/F-LC. Of the fragments screened, **50** (NSC1011) and **51** (NSC1014) appear to have the highest promise as drugs against BoNT/F intoxication [50].

## 3. Cell Wall Enzymes

### 3.1. Glycosidases: N-Acetylglucosaminidases

The peptidoglycan of bacterial cell walls as well as chitins can be hydrolyzed by *N*-acetylglucosaminidases which cut between *N*-acetyl-β-D-glucosamine and many contiguous monosaccharides [51]. *N*-acetylglucosaminidase activity resides in two domains, specifically, the glycosyl hydrolase family 3 (GHF-3; IPR001764) and the glycosyl hydrolase family 73 (GHF-73; IPR001764) [52].

**Figure 9:**
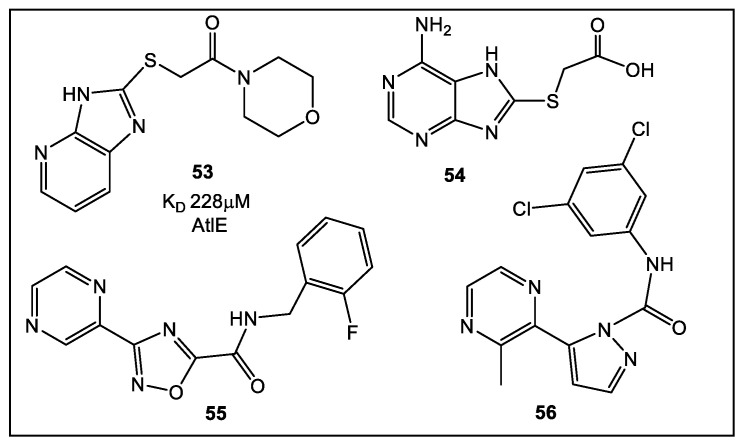
Fragments identified as *N*-acetyglucosaminidase inhibitors. Fragment **53** being the top virtual hit, validated by SPR, with K_D_ of 228 μM for the AtlE [53].

FBLD-based studies have been emerging in the literature for this relatively new antibacterial drug target [53]. Researchers [53] have characterized a library of compounds as inhibitors of the AtlE (enzyme found in *S. aureus*.) subfamily of the aforementioned enzymes. However, these inhibitors have low water solubility. To improve the physico-chemical properties of the initial inhibitor library, a fragment-based library (containing 216, 472 fragments) compiled from several commercial sources was evaluated through virtual screening followed by SPR [53]. From the initial 24 compounds selected, based on the data from the virtual screen, 12 compounds have been validated by SPR. Most of the fragments contain nitrogen heterocycles (di- and tri-azoles) (Figure 9). The best fragment has K_D_ of 228 μM. *N*-acetyglucosaminidase ligands identified were demonstrated to bind different allosteric sites, which may lead to the preparation of antimicrobials that exhibit a novel mechanism of action.

### 3.2. UDP-N-Acetylglucosamine Enolpyruvyl Transferase (MurA)

**Figure 10:**
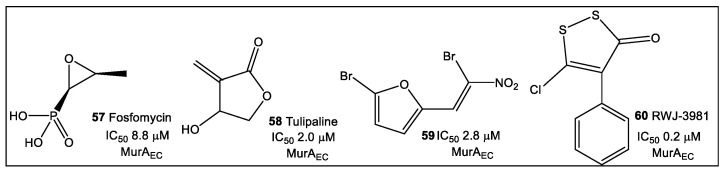
Illustrative examples of small heterocyclic inhibitors of MurA_EC._ They inhibit enzyme activity by forming a covalent bond with cysteine in the enzyme active site.

A library of small electrophilic fragments, predominately nitrogen heterocycles, targeting either one of the Cys (Cys 115 or Cys119) residues in the MurA active site from *S. aureus* and *E. coli*, have also been evaluated as warheads (Figure 10), which are designed to use reactive groups for binding to poorly conserved amino acids, i.e., they are targeted covalent inhibitors [54]. A crucial enzyme targeted in the cytoplasmic biosynthesis of peptidoglycan precursors is MurA. The function of MurA is to catalyze phosphoenolypyruvate (PEP) transfer to UDP-*N*-acetylglucosamine (UNAG), which releases inorganic phosphate [55]. Inactivation of MurA is demonstrated to weaken bacterial cell walls, thus increasing the risk for osmotic lysis [56].

**Figure 11:**
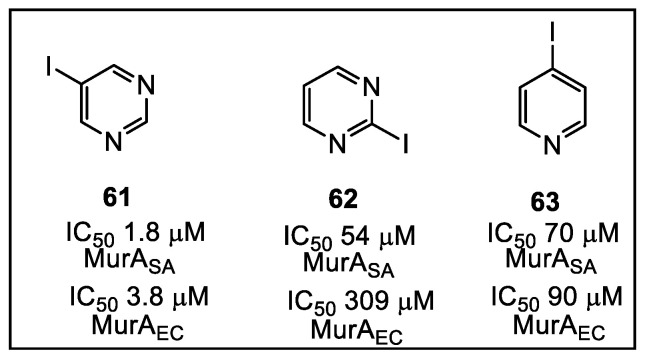
Sulfhydryl active fragments with S_N_Ar have been identified as inhibitors of the active site cysteine of MurA enzymes from *S. aureus* (Mur_SA_) and *E. coli* (Mur_EC_). The fragments, with fragment **61** being the top hit, have been investigated by glutathione stability HPLC and NMR assays, followed by validated by MurA inhibitory assay.

Several representatives of the nitrogen heterocycles, such as **61**–**63** (Figure 11), have demonstrated reversible inhibitory activity against the MurA enzymes of *S. aureus* and *E. coli*. Iodopyrimidine fragment **61** was identified as the best reversible inhibitor of *S. aureus* MurA_SA_, with an IC_50_ of 1.8 μM. The mechanistic studies showed that several fragments such as fragments **61** and **62** reach their maximum inhibitory potency immediately. Some fragments evaluated by the authors of this study (not shown here) are irreversible inhibitors of MurA enzymes.

**Figure 12:**
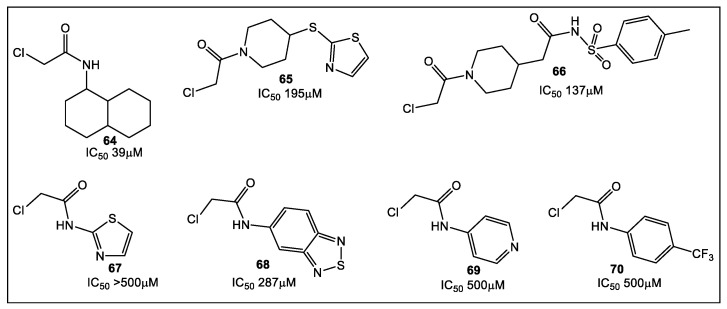
Illustrative examples of chloroacetamide-based covalent inhibitors for MurA from *E. coli* (Mur_EC_) identified by LC-MS/MS and biochemical assays. Fragment **64** was identified as the most effective inhibitor that binds to MurA Cys115. Inhibition of MurA_EC_ by these fragments appears not to be associated with their thiol reactivity, as per evaluation of fragments’ electrophilicity using the Ellman reagent (reduced) as a proxy for cysteine thiol. This suggests the possibility for their further development as inhibitors of the MurA enzymes.

Another sulfhydryl reactive moiety, the chloroacetamide group, has also been incorporated in a library of 47 compounds, the majority of which were synthesized predominantly by a one-step process (Figure 12) [57]. Chloroacetamides were chosen because they show selectivity to different targets, regardless of their electrophilicity being higher than the more common vinylsulfonamides [58]. Most of the chloroacetamide fragments have IC_50_ in the 70–140 μM range. In addition, *E. coli* MurA inhibitors were identified, with the most active inhibitor having a low micromolar IC_50_ [57].

Fragments containing aliphatic rings were determined to be potential inhibitors of MurA, with **64** (containing a primary aliphatic amine) being the most effective inhibitor (39 μM IC_50_). Most heteroaromatic compounds (e.g., **67**–**69**) and aromatic compounds (e.g., **70**) demonstrated either none or at maximum concentration tested (500 μM) a weak inhibition of MurA. Of the aromatic group of compounds, only **68** had activity (287 μM IC_50_).

### 3.3. Phosphopantetheine Adenylyltransferase (PPAT, Also Known as CoaD)

**Figure 13:**
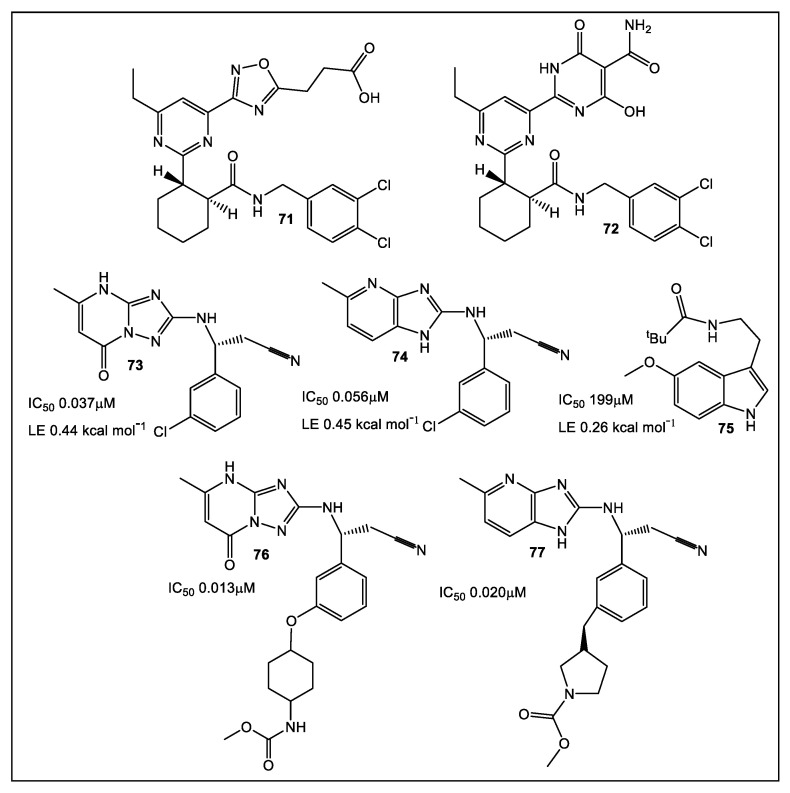
AstraZeneca PPAT inhibitors (**71**, **72**), and from Novartis lead compounds (**72**, **74**), and fragment hit **75**. When fragments co-crystalized with *E. coli* PPAT were examined, **75**, a methoxy tryptamine derivative, partly overlapped with **73**, although its interaction is different from fragment **73**. Fragments **73** and **74** served the basis for development of more than 50 analogs based on with improved on-target potency, including **76**, piperidine carbamate. Analysis of these analogs verified that compounds with an AZ benzimidazole core exhibited an enhanced ability to permeate Gram-negative bacteria. These findings eventually resulted in discovery of compounds, e.g., **77**, with anti-*E. coli* WT activity, which was confirmed by multiple methodologies (biochemical, SPR, and MICs). Regrettably, further progression of this series was halted due to bacterial efflux actions [67].

Coenzyme A (CoA), a cofactor, is essential in the biosynthesis of bacterial membrane lipids, peptidoglycan, teichoic acids, and lipid A [59,60,61]. The next to last step in CoA biosynthesis is catalyzed by PPAT, a hexameric enzyme. While sharing minimal sequence homology with its human ortholog, PPAT shows a high level of sequence homology between bacterial species. This broad spectrum species homology makes PPAT a target for the development of novel antimicrobials [62,63]. Inhibitors of PPAT have been previously reported [64]. These include **71** and **72 (**Figure 13), synthesized by AstraZeneca, which showed both in vitro and in vivo activity against Gram-positive bacteria [65]. Using an FBLD approach, Novartis identified inhibitors of PPAT of Gram-negative bacteria [66,67]. Fragments with activity bind at the *E. coli* PPAT 4′- phosphopantetheine site. With nanomolar IC_50_, lead compounds **73** and **74** (Figure 13) were identified. Unfortunately, **73** and **74** had limited anti-*E. coli ΔtolC* activity [66]. Additional series optimization resulted in inhibitors of *E. coli* WT strain PPAT at picomolar concentrations [67].

## 4. Cell Wall Components: Lectins

**Figure 14:**
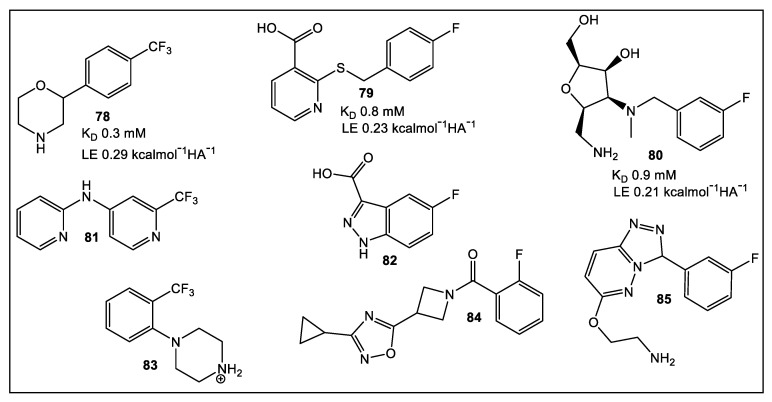
Structures of the best hits after ^19^F NMR screening and validated by SPR with above average of 9% in ^1^H-^15^N TROSY NMR from set of 39 fragments. Fragments **78**, **79**, and **80** demonstrated binding to the secondary binding sites of BambL. The SAR of **78** demonstrated binding pocket identity predicted by computational study of fragment **78** scaffold that is responsible for the binding [70].

Most pathogens adhere to host tissue, either through biofilm formation, or receptor-ligand binding, as part of the colonization process [68]. Lectins, which are carbohydrate-binding proteins, can participate in colonization development since they have a high affinity for mammalian carbohydrates. Therefore, targeting lectins could prove an effective strategy in the prevention and treatment of bacterial and fungal infections [69]. A fragment library of small molecules lacking carbohydrate residues has been developed targeting the propeller lectin BambL from *Burkolederia ambifaria* [70]. This Gram-negative bacterium causes chronic infections and is multidrug resistant. The early inhibitors developed were based on carbohydrate moieties, such as methyl a-l-fucopyranoside (MeFuc; K_D_ = 1 μM) and complex carbohydrates (H type 2 tetrasaccharide; K_D_ = 7.5 μM) [71]. The principal drawback in the use of carbohydrate-based inhibitors is their mass, which restricts their bioavailability [72]. Using a recently developed library of small molecules, researchers focused their effort on fluorinated scaffolds, since they are used as the primary methods for binding studies of the fragments to BambL via ^19^F and T_2_ filtered (CMPG) NMR. They accessed the druggability, i.e., whether a drug discovery project progresses from “hit” to “lead”, of b-propeller lectins of 350 fluorinated fragments via ^19^F and CPMG NMR followed by the computational pocket prediction algorithm SiteMap, SPR, and TROSY (transverse relaxation optimized spectroscopy) NMR [70]. From this analysis, three potential pockets for drug targeting appear present in BambL, the bacterial b-propeller lectin, in addition to possible secondary binding sites in which fragments **78**, **79**, and **80** (Figure 14) could be accommodated. Compounds with the strongest effects in SPR, 19F, and TROSY NMR were assessed by TROSY NMR for a dose-dependent binding. The latter method identified compounds fragments **79**–**85** (Figure 14, the three best being **79**, **80**, and **83**). Fragment **83** showed a two-fold stronger affinity (K_D_ = 0.4 mM) and a better LE value of 0.29 kcalmol^−1^HA^−1^ compared to **79** (K_D_= 0.8 mM, LE = 0.23) and **80** (K_D_ = 0.9 mM, LE = 0.21).

## 5. Bacterial Virulence Factors: DsbA Enzymes of *E. coli and V. cholerae*

### 5.1. EcDsbA

**Figure 15:**
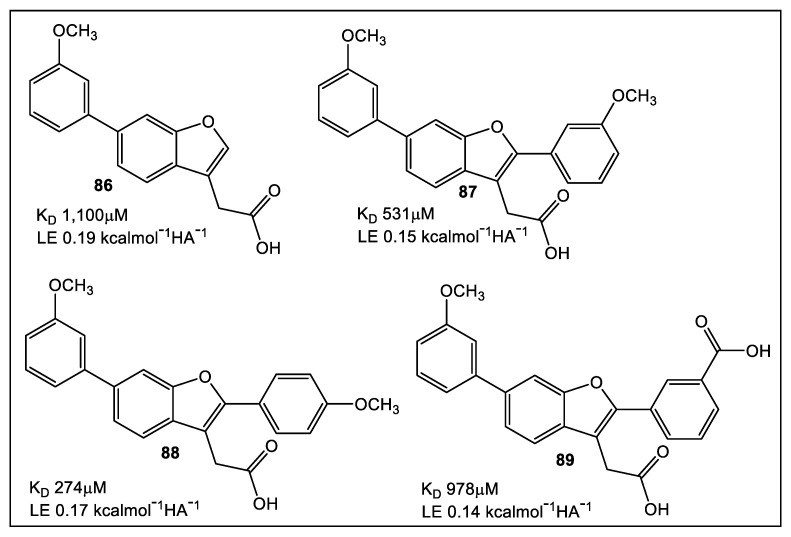
Fragment **86** was identified by NMR and X-ray crystallography as an initial analog for scaffold building. The synthesis started from C-2 to access a more polar region of the binding groove. Confirmation of the appropriateness of C-2 as a starting area for binding pocket access was validated by crystal structural analysis of **87**, **88**, and **89.** Subsequent studies using HSQC showed that relative to parent compounds, C-2 analogues exhibited enhanced K_D_ values. In addition, C-2 analogues inhibited DsbA. This also indicates that there is the potential for development of benzofuran analogues, which could target virulence [73].

FBLD has been developed to aid the efforts on the search for inhibitors with high selectivity for EcDsbA [72]. Initial efforts in developing a fluorine containing FBLD library [73], using a combination of X-ray crystallography and NMR, were performed to characterize the initial non-peptide EcDsbA inhibitors. It was determined, through the use of HSQC NMR titration, that 6-phenoxy and 6-benzyl analogues were the strongest binders [74]. Latter efforts [75] focused on fragments based on the benzofuran scaffold (Figure 15) for evaluation and fine tuning of the binding affinity to the EcDsbA. The synthesis of 2-, 5- and 6-subsituted benzofuran derivatives, along with their structural characterization and in vitro assessment of EcDsbA inhibition confirmed the high affinity of the benzofuran analogs for EcDsbA with improved in vitro inhibition.

### 5.2. VcDsbA

**Figure 16:**
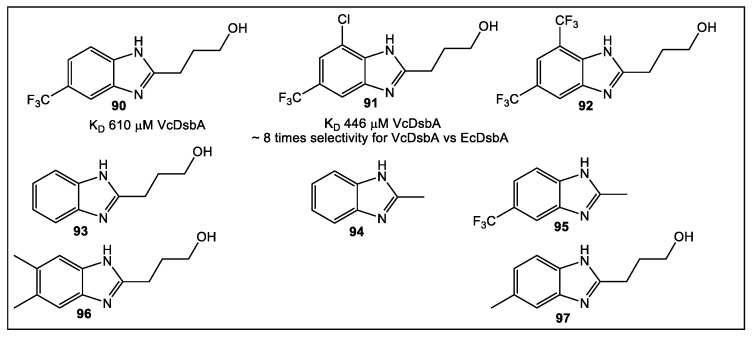
Structures of fragment **90** analogues screened by ^1^H-^15^N HSQC NMR. To elucidate the differences in ligand binding of fragment **91** to VcDsbA vs. EcDsbA, crystal structures were obtained of each, complexed with sodium taurocholate, a known ligand. Crystallography showed that sodium taurocholate exhibits binding orientations in complex with VcDsbA that differ from that which occurs with EcDsbA. In addition, the protein–ligand interactions resulting in stabilization of binding orientations are revealed [76].

Another use for FBLD has been to identify novel leads built on the benzimidazole scaffold. Ideally, these leads would bind (K_D_ of 446 μM) to oxidized VcDsbA via its hydrophobic groove. Interestingly, this benzimidazole fragment has an ~eight-fold selectivity for VcDsbA over EcDsbA. In addition, it has the capability of binding to oxidized EcDsbA, (K_D_ > 3.5 mM) (Figure 12) [76]. Starting with 500 commercially available fragments, using STD NMR analysis, 15 fragments were identified as hits [76]. The binding of these 15 fragments was confirmed in the presence and absence of each fragment by recording ^1^H-^15^N HSQC spectra of VcDsbA. Fragment **90** (Figure 16), built on the benzimidazole chemotype, had the best affinity for binding in the hydrophobic grove of VcDsbA according to the ^1^H-^15^N HSQC NMR perturbation in chemical shift. Therefore, fragment **90** was chosen for further SAR studies. To identify the area(s) of fragment **90** responsible for binding to the VcDsbA, seven fragment **90** analogs were synthesized. Of these seven compounds, fragment **91** proved to have the best affinity for the VcDsbA, and it was chosen for further investigation (Figure 16). Despite the weak binding affinity of **91**, in a complex with VcDsbA, the NMR model offers a structural basis for its selectivity and provides a prototype for ongoing fragment development [76].

## 6. Quorum Sensing—QS

**Figure 17:**
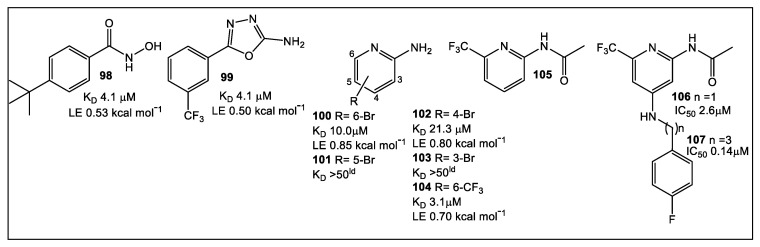
Optimal starting point identification using thermodynamic profiling of fragment sized PqsR ligands. A re-evaluation of the **98**, **99**, and **100** fragment scaffolds showed that **100** has remarkably improved enthalpic efficiency (EE) and ligand efficiency (LE) values. Hit **100** led to **104** optimization resulting in **106**, which enabled further optimization through flexible fragment growing. The *N*-propyl amine linker in **107** shows an increase in potency (20-fold). Crystallography of **107** in complex with PqsR91-319 reveals the extended linker pointed further into the pocket containing the alkyl chain of the natural ligand.

Virulence factor expression can be controlled by chemical signaling molecules during the process of quorum sensing (QS) [77]. FBLD has been applied in the discovery and optimization of 2-aminopyrimidine QS inhibitors, one of the four QS systems employed by *Pseudomonas* species. The *Pseudomonas* Quinolone Signal (PQS) system was focused on since it is specific to *Pseudomonas* spp. and *Burkholderia* spp. [78]. The PQS system employs alkylquinolones (AQs), rather than the widespread Gram-negative bacterial signaling molecules *N*-acylated homoserine lactones (AHLs). This system, a transcription regulator, controls production of virulence factors including elastase, pyocyanin, and lectins [79,80]. The first inhibitor of PqsR, which was based on the natural ligand HHQ, was modified upon [78] to improve its poor physicochemical profiles using SPR technology with two fragment screenings. This approach resulted in the identification of hydroxamic acid **98** and the 2-amino-oxadiazole **99** (Figure 15) as PqsR inhibitors. However, further attempts to expand the two structures were unsuccessful, since these analogs lack activity against *P. aeruginosa*. Application of an enthalpic efficient approach led to fragment **100** (Figure 17). This was followed by introducing a flexible linker in fragment **105** (Figure 17), leading to compound (**106**, Figure 17) which completely inhibited pyocyanin (**107**, Figure 17) in *P. aeruginosa* at nanomolar concentrations. Additional information on recent developments of lead compounds based on FBLD targeting *P. aeruginosa* is summarized in a recent comprehensive review [81].

## 7. Bacterial Enzymes Catalyzing Purine Synthesis

### 7.1. Mycobacterium Abscessus Phosphoribosylaminoimidazole Succinocarboxamide Synthetase (PurC or SAICAR Synthetase)

**Figure 18:**
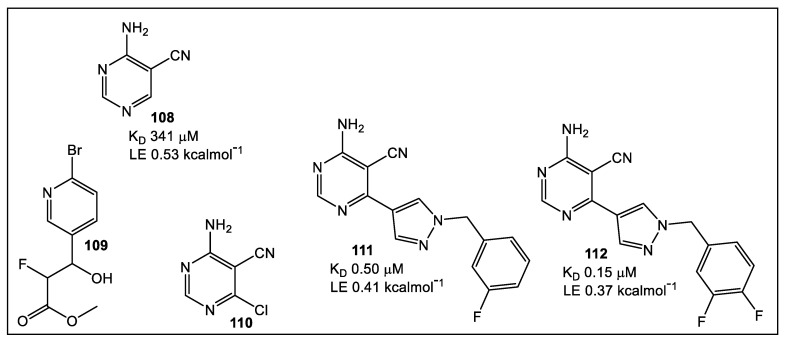
Fragment **108** demonstrated the highest ligand efficiency (LE). Fragment **108** with MabPurC revealed an interaction at the 6-postion of the pyrimidine in the “ribose binding pocket”. This binding position appeared to be tolerated since it also was functional **110** binding. Fragment expansion here permits the addition of the pyridine moiety of fragment **109** into fragment **108.** However, the introduction of a flexible linker was necessary for linkage of the fragments since they were almost perpendicular to each other. Synthesis of a library of a dozen compounds, where different linkers were explored, led to identification of compound **112** having the best binding affinity and LE [89].

An enzyme essential in bacterial and fungal purine biosynthesis is PurC [82,83,84]. Recently, PurC has been examined as a potential drug target. Specifically, the PurC of *Mycobacterium abscessus* (Mab) has been the focus since it proves difficult to treat, particularly in individuals with cystic fibrosis. The fact that the bacterial PurC and its human analog PAICS exhibit significant differences, both structurally and functionally, makes PurC a reasonable target for antimicrobial drug development [85,86,87,88]. The FBLD methodology resulted in identification of a new inhibitor class based on 4-amino-6-(pyrazol-4-yl)pyrimidine [89]. This strategy utilized hits detected by high throughput X-ray (XChem, Diamond Light Source) from several fragment libraries (1853 total fragments screened) as starting points for further development [90]. The fragments’ potential as inhibitors of PurC in Mab (MabPurC) were validated via different methodologies including differential scanning fluorimetry (DSF), isothermal titration calorimetry (ITC), and X-ray crystallography. Furthermore, these findings demonstrated that MabPurC is essential for Mab, thus justifying this approach for continuing the development of specific inhibitors [89]. Eight fragments were selected for further derivatization. Fragments identified by X-ray crystallography or XChem screening, respectively, to be in complex with MabPurC were **108** and **109** (Figure 18). The derivatization of both fragment **108** and fragment **109** produced a little over 30 analogs, from which compound **111** (Figure 18) showed an inhibition of MabPurC in the nanomolar range and a very good LE. Compound **112** (Figure 18) demonstrated the best inhibition and LE for MabPurC [89].

### 7.2. tRNA (m1 G37) Methyltransferase (TrmD) from Mycobacterium Abscessus (MabTrmD)

**Figure 19:**
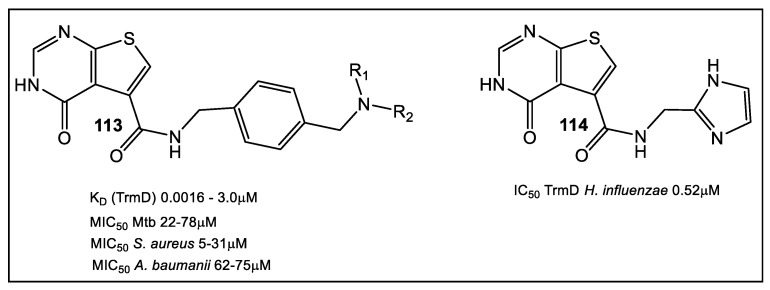
Inhibitor **113** of TrmD of Gram-positive, Gram-negative bacteria, and Mtb developed using P. *aeruginosa* and Mtb TrmD crystal structures with a series of thienopyrimidinone derivatives identified through high-throughput screening with nanomolar potency against TrmD [97]. Compound **114** was developed when FBLD approach to develop selective inhibitors was applied against *H. influenzae* TrmD [99].

TrmD, a member of the SpoU-TrmD (SPOUT) RNA methyltransferase family, was evaluated as a drug target using FBLD [91]. The methyl transferase TrmD, like MabPurC, is essential for viability in Gram-positive bacteria, e.g., *S. aureus*, Gram-negative *P. aeruginosa,* as well Mtb and Mab [92,93,94,95,96]. Validating TrmD as a drug target was accomplished initially by high-throughput screening against *P. aeruginosa* TrmD in order to identify low-micromolar inhibitors [97], followed by in vivo mechanism testing (**113**, Figure 19) [98]. In addition, FBLD was used to develop selective inhibitors that ordered the interdomain linker, such as that of tRNA for *Haemophilus influenzae* TrmD (**114**, Figure 19) [99]. Unfortunately, the compounds displayed little activity against Gram-positive and Gram-negative pathogens, e.g., *E*. *coli* efflux mutants and *H. influenzae* (Figure 19) [99]. The scanning fluorimetry (DSF) primary screen, then thermal shift cut-off value (3 S.D. from the negative control) FLBG approach were used for MabTrmD hit optimization [91], screening an initial fragment library of 960 fragments.

**Figure 20:**
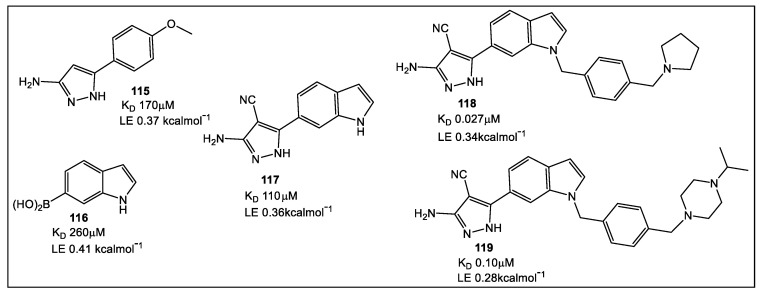
Fragment hits for MabTrmD. Elaboration on fragment **117** resulted in **118** (K_D_ 27 nM, LE 0.34), a low-nanomolar affinity compound with anti-MabTrmD activity. Screening of merged fragments **115** and **116** (e.g., **118**) against Mab and Mtb showed promising MIC values. Compound **119** had the best MIC values against Mtb and Mab of 1.6 μM and 2.3 μM in supplemented 7H9 medium [91]. This series of compounds had activity against mycobacteria in vitro and in vivo [97].

The X-ray studies of fragment hits led to 27 fragments which bind at the Mab TrmD SAM binding pocket. Using the fragment-merging strategy, compounds were further developed to increase affinity binding for MabTrmD (< four-fold) then screened for in vitro anti-Mab activity in both culture and a human macrophage infection model [91]. Several compounds (Figure 20) displayed activity against Mtb [93]. Fragments, **115** (K_D_ 170 μM, LE 0.37), and **116** (K_D_ 260 μM, LE 0.41), as well as compound **117** (K_D_ 110 μM, LE 0.36) showed increased affinity (Figure 20).

### 7.3. Mycobacterium Thermoresistible (MthIMPDH) Inosine-5′-Monophosphate Dehydrogenase (IMPDH) 

**Figure 21:**
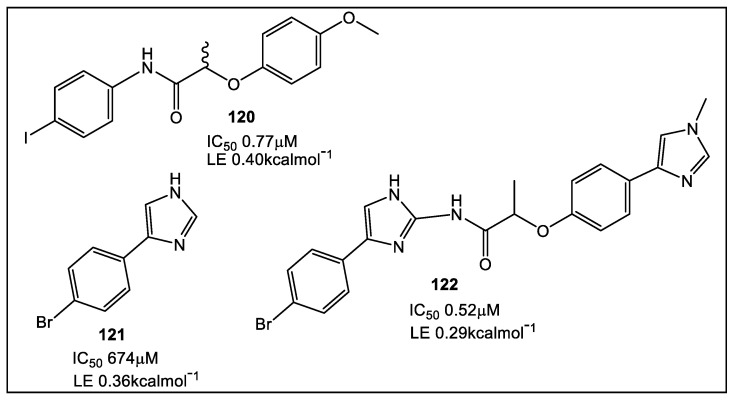
Fragment-linking strategy led to synthesis of 18 compounds based on fragments **120** and **121**. Compound **122** demonstrated markedly improved MthIMPDH ΔCBS inhibition (LE 0.29; 0.52 μM IC_50_), which compared to **120** and **121** is significantly more potent (1300-fold); compound **121** X-ray structure with shown MthIMPDH at lower right. The racemate **122** has ~50% the Mth IMPDH ΔCBS inhibition as compared to its (*S*)-isomer, a pattern similar to that previously reported for other IMPDH inhibitors [109,110,111]. However, anti-Mtb H37Rv activity of the most potent analogues (0–100 μM tested) lacked clinically relevant activity (MIC_90_ ≥ 50 μM). This lack of activity may be the result of poor cell permeability, metabolic instability, and/or efflux [109]. Additional information about FBLD developments in the search for anti-Mtb compounds can be found in these recent reviews [112,113].

The first unique step in the synthesis of guanine nucleotides is catalyzed by IMPDH (Figure 1) [100]. This enzyme has been targeted in the development of immunosuppressive [101], anticancer [102,103], and antiviral drugs, and now antimicrobials, including Mtb [104,105,106,107,108,109]. In addition, FBLD was utilized for developing inhibitors for another mycobacterial enzyme, the IMPDH from *Mycobacterium thermoresistible*, MthIMPDH [109]. Analogous to the screens used against the Mab enzymes, the screen against MthIMPDH involved the fragment library of 960 fragments. From fragment hits from biochemical assays (18 fragments), 6 were studied by X-ray crystallography. These data suggested an approach for optimization via fragment-linking being most suitable for synthetic modifications, namely fragment hits **120** and **121** (Figure 21) [109].

### 7.4. Threonyl-tRNA Synthetase from Salmonella Enterica

**Figure 22:**
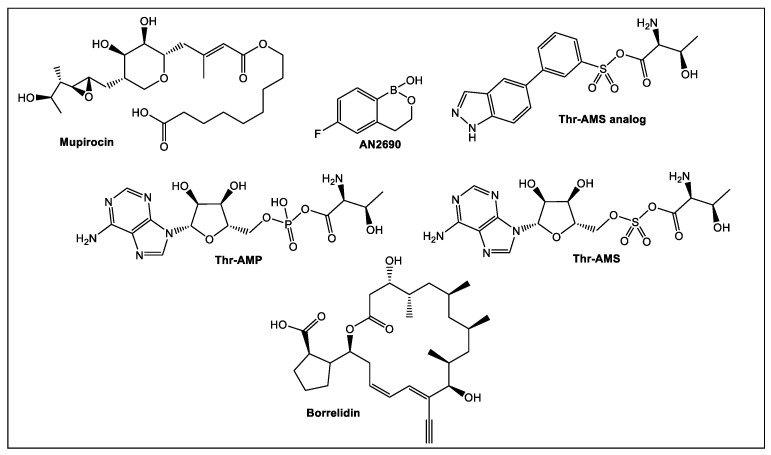
The intermediate product Thr-AMP of ThrRS. Representative inhibitors of ThrRS in the nanomolar range.

**Figure 23:**
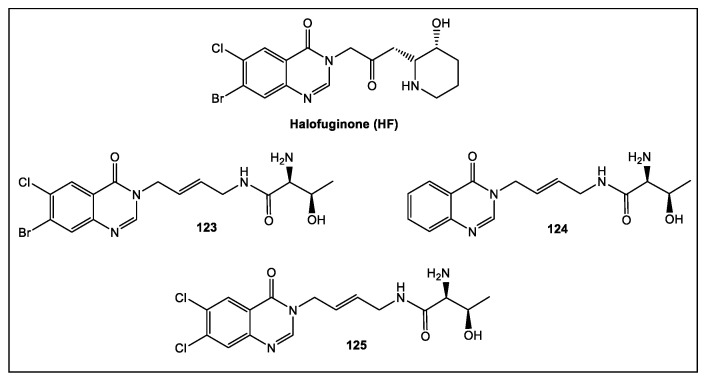
Halofuginone (HF), an inhibitor of the plant analog of ThrRS, the prolyl-tRNA synthetase (ProRS). Fragments **123** and **124** are the best representatives of a series of synthetic analogs of HF. Compound **125** demonstrated antibacterial activity (MIC 16 mg/mL) against *E. coli* and *Salmonella enterica* [115].

A crucial member of the aminoacyl-tRNA synthetases (aaRS) family is threonyl-tRNA synthetase (ThrRS) which catalyzes amino acids attachment to their tRNAs. Inhibition of ThrRS has potential utility in the treatment of infections and cancers. Several of the currently known inhibitors are shown in Figure 22. The three substrates of ThrRS, i.e., tRNAThr, ATP, and L-threonine interact with three corresponding pockets on the ThrRS catalytic domain [114]. The usual binding sites for the known aaRS inhibitors are the amino acids’ binding sites and/or ATP. Other active sites of ThrRS have been explored recently by utilization of FBLD to develop new aaRS-based inhibitors. These ThrRS dual inhibitors are based on the chlorinated analog halofuginone (HF) (Figure 23) of the natural product febrifugine [115]. The latter is isolated from *Dichroa febrifuga* Lour, a medicinal plant used in traditional Chinese medicine to treat malaria [115]. HF has been demonstrated to be an inhibitor of the plant analog of ThrRS, the prolyl-tRNA synthetase (ProRS). Its binding to ProRS differs from most of the inhibitors shown in Figure 23, since HF binds simultaneously to the ThrRS L-threonine and tRNAThr binding pockets which increases selectivity and activity [116].

Thr-AMS, a non-hydrolyzable synthetic derivative of Thr-AMP [117], and its simplified Thr-AMS analog improved specificity and activity [116]. Mupirocin is another inhibitor of aaRSs, i.e., bacterial isoleucyl-tRNA synthetase (IleRS). This naturally occurring antibiotic is used clinically against methicillin-resistant *S. aureus* (MRSA) and others [118,119]. AN2690, another FDA approved inhibitor, binds to the eukaryotic leucyl-tRNA synthetase (LeuRS) editing site and is clinically used in the treatment of onychomycosis [120,121]. Another naturally occurring inhibitor of ThrRS is borrelidin, an 18-membered polyketide macrolide isolated from *Streptomyces* species [122], which competes with all three substrates (tRNAThr, ATP, and L-threonine) and has anti-bacterial, anti-fungal, and anti-cancer activity in the nanomolar range [123].

HF occupies the adenosine 76 (A76) and L-proline binding pockets via its halogenated quinazolinone and hydroxypiperidine ring, respectively, to inhibit ProRS [124,125]. HF activity is enhanced by ATP, usually reaching millimolar concentrations in vivo [126,127]. Thus, the inhibitory mechanism of HF presents an attractive avenue for drug development. An HF analogue series were designed and synthesized as ThrRS inhibitors by utilizing the FBLD approach [115].

When anti- *E*. *coli* activity of the compounds was determined, **123** and **124** demonstrated the best activity. Although **123** lacked activity against Gram-positive bacterial species (*S. aureus*, MRSA, *Enterococcus faecalis*) and *P. aeruginosa,* **125** exhibited activity against *E. coli* and *S. enterica* 87 (16 mg/mL MIC). Crystal structures of SeThrRS (ThrRS of *S. enterica* 87) with or without **125** indicated that it interacted with the tRNA binding pocket of SeThrRS via a dual-site induced-fit mechanism. However, more studies are needed to improve tRNA-amino acid dual-site inhibition [115].

## 8. Primase/SSB-Ct Interaction

**Figure 24:**
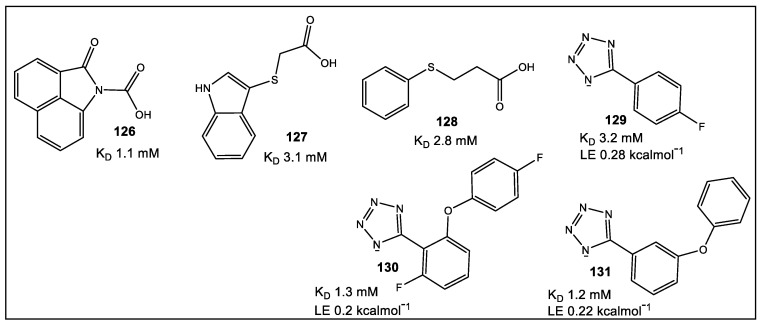
Hits chosen for further optimization based on the STD-NMR and their binding affinities (K_D_ values; ^15^N^–1^H HSQC NMR titration).

Bacterial DnaG primase is involved in short RNA primer synthesis, functioning during chromosomal replication to initiate chain extension by replicative DNA polymerase(s). DnaG of *E. coli* interacts with several proteins including SSB, an ssDNA-binding protein. SSB is an interaction hub binding >14 proteins participating in DNA replication, repair, and recombination [128,129]. FBLD was utilized to evaluate screening of 1140 structurally diverse fragments [130] by STD-NMR and SPR assays that detected primase/SSB-Ct interaction inhibitors [131]. SPR competition assay initially identified six fragments able to compete with immobilized SSB-Ct peptide. The SPR hits, as well as combinations incorporating the MIPS library (1140 fragments) [130] were analyzed by STD-NMR [131]. Of the screened fragments, 56 exhibited strong intensity difference with an additional 62 showing moderate intensity difference [130]. The concluding STD-NMR of 80 fragments identified ~50 fragments for exclusion [130]. Fragments 126–129 (Figure 24) which had binding affinities in the 1–3 mM range, as determined by NMR titration experiments, were tested further.

Based on the assumption that the tetrazole moiety in **129** would increase lipophilicity and improve membrane permeability, as compared with **126**–**128**) [131], **129** was chosen for further optimization. Although in silico screening identified tetrazole analogs (10) with potentially advantageous binding poses vis a vis SSB-Ct peptide, only **130** showed any binding to DnaGC with a three-fold increase as compared to **129** (15N–1H HSQC spectra; K_D_ = 1.3 mM) [131]. The only available analogue with structural similarity to the ligands **5** (ZINC database) was found to be **131** (Figure 24) [132]. Based on chemical shift perturbation (CSP) modeling studies, **130** and **131** form electrostatic and hydrogen bond complexes in the binding pockets. A combination of binding to the C-terminal domain of DnaG primase as well as SSB-interacting is a promising start for the development of drugs with long-lasting potential.

## 9. Fragment-Based Lead Discovery (FPLD); Cell-Based Screens for the Identification of Microbial Inhibitors of *Leishmania*, *Plasmodium falciparum*, *Neisseria*, *Mycobacterium*, and Flaviviruses

**Figure 25:**
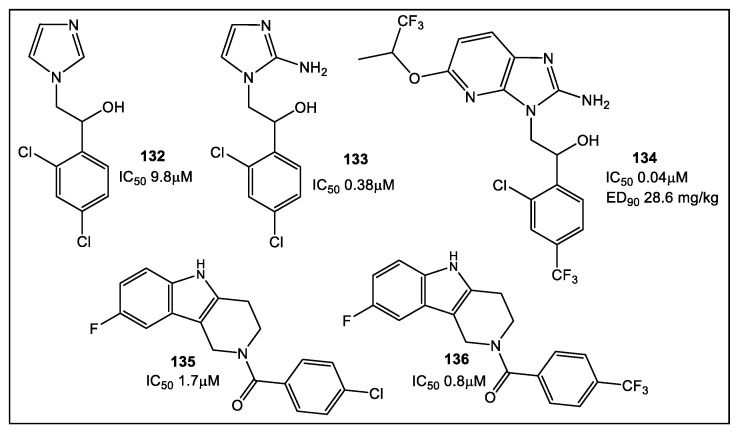
Best fragment hits against *P. falciparum*, **132** from a library of 1604 fragments and **133** and **134** from Astra Zeneca HTS screened 500,000 fragment corporate library. Tetrahydro-β-carboline compounds **135** and **136** from a 1604 fragment library against *N. meningitidis*.

Multiple methodologies have been utilized to test fragments as potential antimicrobials in clinically appropriate environments. However, few fragments’ screens were conducted without any preconceived expectations relative to the putative mode of action [133,134,135]. The potential advantage of FBLD is that it is a target-based approach wherein lipophilicity and selectivity can be controlled for relative to specific targets. There are successful examples of optimization strategies, such as LE in the area of antimicrobial lead development, through which a lead fragment is developed into a drug with clinical potential [136]. The useful metric of LE becomes less of a gold standard when MICs are the principal gauge of antimicrobial activity. In order to get around the issue of optimization of a lead compound as a potential antimicrobial drug candidate, after optimization of the initial fragment hit activity and efficacy against a specific antimicrobial drug target, FPLD was applied to screen *M. tuberculosis* to identify compounds that exhibit favorable drug properties in a whole cell screen [130] and mouse models [134]. In addition, a whole cell screen has been used for inhibiting *Leishmania* parasites [135]. FPLD, however, poses a challenge in determining a SAR when a specific target has not been identified, since MIC activity could be the result of off-target effects. This FPLD screen against *Leishmania* [135] has been expanded to utilization of FPLD against a variety of microorganisms, such as *Plasmodium falciparum*, *Neisseria*, *Mycobacterium*, and flaviviruses [137]. Several illustrative examples of fragments with activity against *P. falciparum* and *Neisseria meningitidis* are shown below (Figure 25) [137]. The fragments with activity against *P. falciparum* identified through a phenotypic screen [137] have provided an interesting example of fast and effective application of FPLD at the start for lead identification. Fragment hit **132** (Figure 25) from a library of ~1600 compounds [137] has high structural similarity and comparable activity to hit **133** (Figure 25) which has been identified from a library of 500,000 compounds, and later optimized to hit **134**, with activity in the nanomolar range. This example implies that fragment libraries used together with phenotypic screens have the capability to detect hits that can be exploited to obtain clinically relevant activity and concentrations. Phenotypic screening could be an accessible alternate approach for the identification of new antimicrobial leads at the start of an FBLD, especially in institutions with limited resources.

## 10. Conclusions

Different approaches to screening for antibacterial leads continue to be employed-from natural product-inspired scaffolds and their derivatization, to making use of the de novo molecular design program SPROUT [24,27] for whole cell fragment screening [137]. In the case of β-lactamase boronate leads [21], it was fortunate that FLBD-guided modifications improved compounds’ target affinity, which also resulted in whole cell activity. Moreover, QPX7728 is now considered as an ultrabroad-spectrum inhibitor of serine and metallo-β-lactamases. Regretfully, this, as has been seen in many other antibacterial projects, is rarely the case. The latter might be a good reason to prompt the investigation of the activity of more advanced fragments, whose good target specificity and promise for antibacterial activity have been determined solely based on purified protein, to be evaluated by a whole cell assay. This appears to be the important strategy utilized for the for successful optimization of the cyclic boronates as antibacterial candidates, e.g., compound QPX7728 [23].

## 11. Future Direction

Recent developments using fragments to probe-identified unique microbial targets may start to address the challenges associated with antimicrobial resistance. In addition, the identification of new drug targets by using fragments in a phenotypic screen has the potential for opening novel avenues for fragment weaponization. Furthermore, these fragments could be doubly functionalized, as has been demonstrated in attempts to expand the pharmacopoeia of anti-cancer drugs that can be used in mammalian systems [138,139]. However, since this approach presents a significant challenge in screening small-molecule libraries, CRISPR/Cas9 technology may offer some helpful alternatives, particularly if it could be adapted to high throughput settings [140]. This gap in identification of novel ligands and targets could also be filled by the use of fully functionalized fragments (FFFs) together with covalent fragment screening, which are nicely summarized in recent reviews [141,142]. Thus, it may be that the primary phenotypic screens are what will guide the “rational” fragment-based discovery of novel antibiotics in the next several years.

## Data Availability

Not applicable.
